# Draft Genome Sequence of *Gulbenkiania indica* Strain HT27^T^ (DSM 17901^T^) Isolated from a Sulfur Spring in India

**DOI:** 10.1128/genomeA.00830-16

**Published:** 2016-08-11

**Authors:** Jhasketan Badhai, Kunwar Digvijay Narayan, William B. Whitman, Subrata K. Das

**Affiliations:** a Department of Biotechnology, Institute of Life Sciences, Nalco Square, Bhubaneswar, India; b Department of Microbiology, University of Georgia, Athens, Georgia, USA

## Abstract

*Gulbenkiania indica* strain HT27^T^ was isolated from a sulfur spring. Here, we report the first representative draft genome sequence of a type strain of the genus *Gulbenkiania*. The estimated genome is 2.8 Mb, with 2,713 protein-coding sequences.

## GENOME ANNOUNCEMENT

*Gulbenkiania indica* strain HT27^T^ is a Gram-negative, strictly aerobic rod and motile bacterium belonging to the class *Betaproteobacteria*. The bacterium was isolated from a sulfur spring from Athmallik, Orissa, India (1). Analysis of the whole genome will provide insight into the bacterium’s ecological functions and evolution.

The draft genome of *Gulbenkiania indica* strain HT27^T^ was generated at the DOE Joint Genome Institute (JGI) using the Illumina HiSeq 2000 platform (2). An Illumina standard shotgun library was constructed and sequenced using the Illumina HiSeq 2000 platform, which generated 9,623,172 reads totaling 1,453.1 Mb. The filtered Illumina reads were assembled using the Velvet (3), wgsim (https://github.com/lh3/wgsim), and Allpaths-LG (4) tools. The final draft assembly contains 23 contigs in 20 scaffolds, totaling 2.8 Mb, with an input read coverage of 258.1-fold. The largest and *N*_50_ contigs are 734.0 kb and 286.1 kb, respectively, with a G+C content of 63.0%.

The genome was annotated using the JGI Microbial Genome Annotation Pipeline (5). Genes were identified using the Prodigal (6) and GenePRIMP (7) programs. The tRNA, rRNA, and other noncoding RNA genes were identified by searching the genome using the tRNAscan-SE tool (8), rRNA gene models built from SILVA (9), and INFERNAL (http://infernal.janelia.org), respectively. The draft genome sequence has 2,713 coding sequences (CDSs), 16 pseudogenes, 59 tRNAs, 17 rRNAs and 10 other RNAs.

### Accession number(s).

This whole-genome shotgun project has been deposited at DDBJ/EMBL/GenBank under the accession no. LION00000000. The version described in this paper is the first version, LION01000000.
